# The HIV-1 gag p6: a promising target for therapeutic intervention

**DOI:** 10.1186/s12977-024-00633-2

**Published:** 2024-01-23

**Authors:** Xiaowei Chen, Xiao Wang

**Affiliations:** 1 School of Basic Medical Sciences, Binzhou Medical University, 264003 Yantai, China; 2 Medicine & Pharmacy Research Center, Binzhou Medical University, 264003 Yantai, China

**Keywords:** HIV-1 Gag p6, Viral replication and budding, Antiretrovirals

## Abstract

The p6 domain of the Gag precursors (Gag p6) in human immunodeficiency virus type 1 (HIV-1) plays multifunctional roles in the viral life cycle. It utilizes the endosomal sorting complex required for transport (ESCRT) system to facilitate viral budding and release from the plasma membrane through the interactions with the ESCRT-I component tumor susceptibility gene 101 (TSG101) and with the ALG-2 interacting protein X (ALIX). Moreover, Gag p6 contributes to viral replication by a range of posttranslational modifications such as SUMOylation, ubiquitination and phosphorylation. Additionally, Gag p6 also mediates the incorporation of the accessory protein Vpr into virions, thereby promoting Vpr-induced viral replication. However, less attention is focused on Gag p6 as therapeutic intervention. This review focuses on the structures and diverse functions of Gag p6 in viral replication, host cells, and pathogenesis. Additionally, several challenges were also discussed in studying the structure of Gag p6 and its interactions with partners. Consequently, it concludes that the Gag p6 represents an attractive target for the development of antiretroviral drugs, and efforts to develop p6-targeted antiretrovirals are expected to undergo significant growth in the forthcoming years.

## Introduction

The human immunodeficiency virus type 1 (HIV-1) consists of three main structural polyproteins, namely Gag, Pol, and Env, which serve as crucial structural components of the virus. Among these, the Gag polyprotein plays a vital role in the viral assembly and budding, which undergoes cleavage into several structural components and two spacer peptides by the viral protease, including matrix (MA), capsid (CA), nucleocapsid (NC), p6, as well as SP1 and SP2. These processing events are crucial for the viral maturation [1, 2]. Within the Gag polyprotein, the p6 domain of the Gag (Gag p6) consists of 52 amino acids. Despite its small size, Gag p6 plays multiple roles in the viral life cycle, host cells, and pathogenesis. In the absence of Gag p6, viral particles accumulate at the cell membrane, leading to an incomplete infection cycle [3]. This paper reviewed the structures and multifunction of Gag p6 during the viral life cycle and also discussed the potential of Gag p6 as an attractive target for the development of antiretroviral drugs.

## Structures of HIV-1 Gag p6

**Fig. 1:**
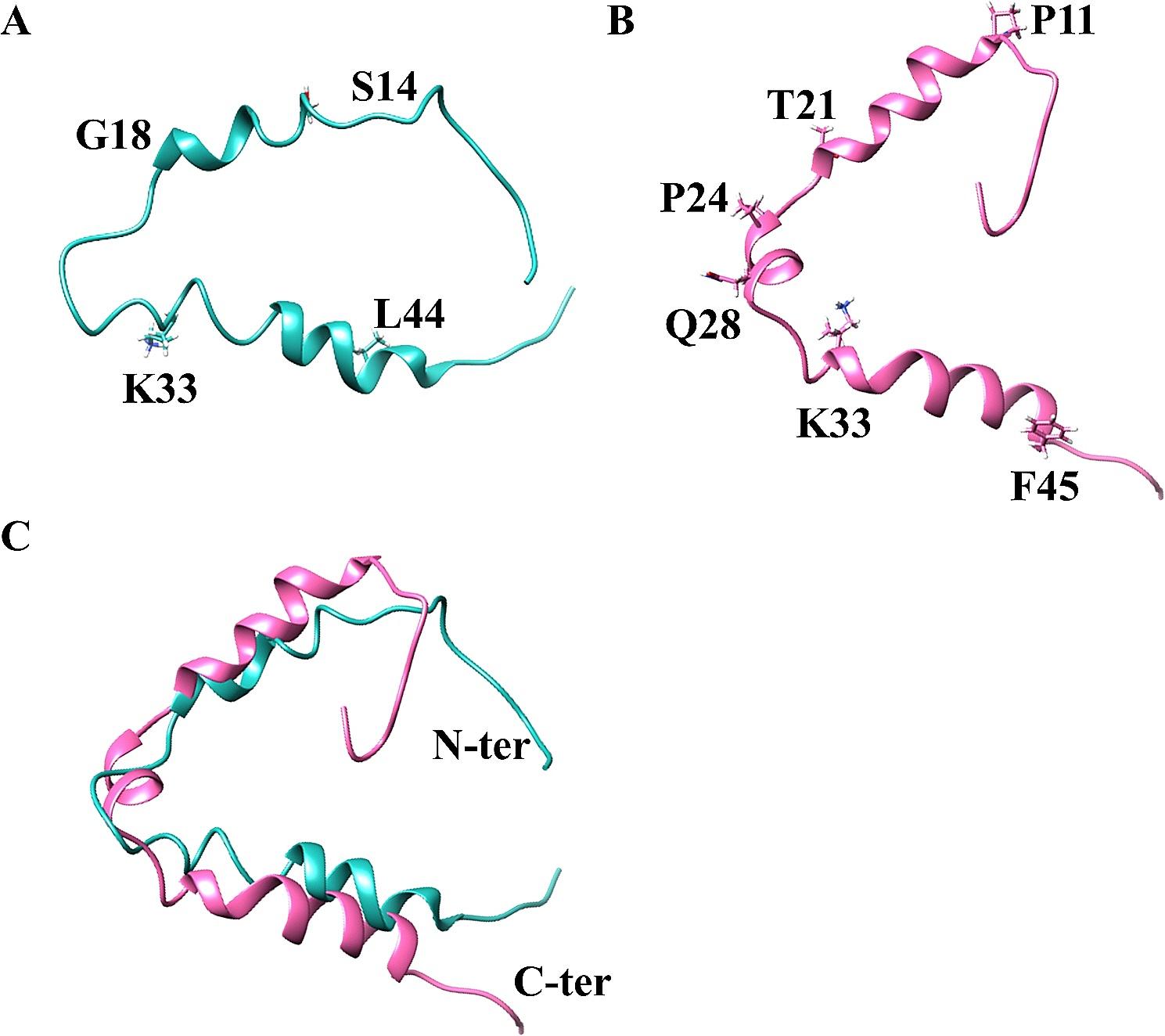
Structures of HIV-1 p6 in different solutions. Solution structures of HIV-1 p6 (**A**) in a distilled trifluoroethanol-*d*_2_ (TFE-*d*_2_)/H_2_O environment (PDB: 2C55) and (**B**) in the presence of dodecylphosphocholine (DPC) micelles (PDB: 7P3O). (**C**) Superposition of two structures

The HIV-1 assembly occurs at the plasma membrane of the infected cell through the oligomerization of Gag polyprotein, which forms a hexameric protein lattice [4–6]. Although the structural characteristics of most components of the Gag polyprotein have been extensively studied, the available information on Gag p6 structure remains limited due to its inherent high flexibility. To overcome this limitation, the free p6 as its mature form was chemically synthesized and its structure was determined by solution nuclear magnetic resonance (NMR) in a distilled trifluoroethanol-*d*_2_ (TFE-*d*_2_)/H_2_O environment [7]. The results revealed that the free p6 adopts a flexible helical structure, consisting of two short helices spanning residues 14 to 18 and 33 to 44 (Fig. 1A). The helices are flanked by flexible N- and C-terminal domains. By contrast, other studies showed the free p6 comprises two well-defined helical structures at N- and C-terminals, connected by a flexible hinge region in a 100 mM dodecylphosphocholine (DPC) micelle solution [8]. Our studies revealed an additional small α-helix in HIV-1 p6, spanning residues 24 to 28 (Fig. 1B), in a 300 mM DPC micelle solution (to be published, PDB: 7P3O), as compared to its structure in a TFE-*d*_2_ solution (Fig. 1C). These findings indicate that Gag p6 or the free p6 may have different conformations depending on the hydrophobic environment in which they are present. Interestingly, the interactions between Gag p6 and its partners occur in different cellular environments, such as the cell membrane and cytoplasm. Consequently, depending on its specific environment within the cell, Gag p6 adopts different conformations to exhibit diverse functions.

## The role of Gag p6 in viral budding

The endosomal sorting complex required for transport (ESCRT) system plays a key role in cellular abscission, multivesicular body biogenesis as well as viral budding. This system consists of five cytosolic protein complexes known as ESCRT-0, ESCRT-I, ESCRT-II, ESCRT-III, and Vps4-Vta1, along with accessory protein ALG-2 interacting protein X (ALIX) [9]. In the process of the delivery of multivesicular bodies, the ESCRT system is recruited to the cargo by recognizing the ubiquitylation signals, whereby it acts as a cargo recognition and membrane deformation machine. During this process, tumor susceptibility gene 101 (TSG101), a component of ESCRT-I, and ALIX primarily coordinate the initial stages of membrane trafficking and abscission within the cells. The ubiquitin moiety on the cargo is recognized by TSG101 and ALIX. Following cargo recognition, ESCRT-I orchestrates the sealing of the membrane compartment through subsequent interactions with ESCRT-II and ESCRT-III, while ALIX directly recruits ESCRT-III. The recruitment of ESCRT-III is a dynamic process that occurs in a temporally and spatially regulated manner within the cells, of which ESCRT-III forms filaments, consequently resulting in the scission and sealing of cellular membranes from the nuclear envelope to the plasma membrane or other membranes [10].

**Fig. 2:**
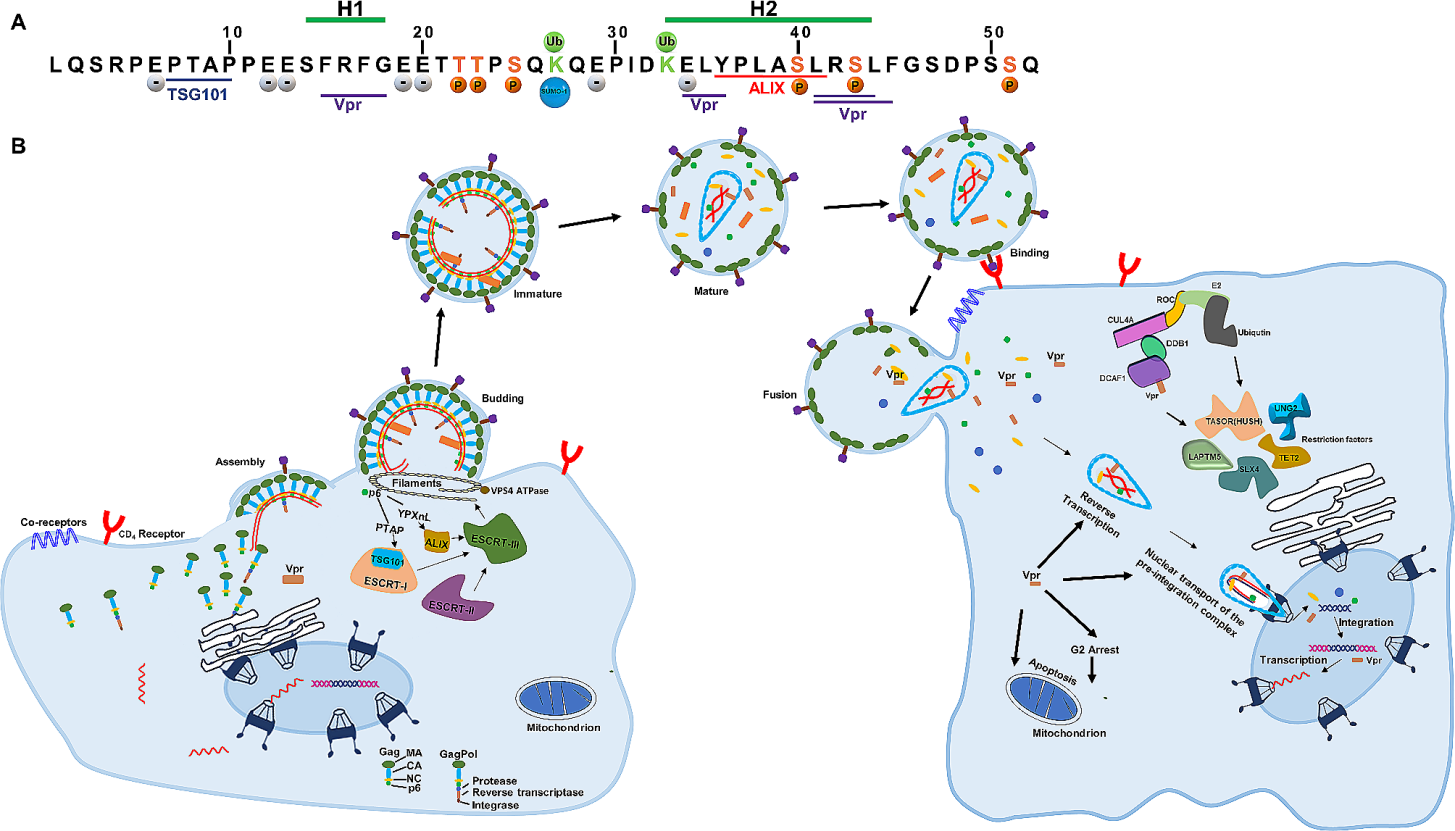
Functional sites of HIV-1 Gag p6, process of viral budding, and Vpr multifunction in the newly infected cells. (**A)** Summary of the primary sequence and important functional sites in HIV-1 Gag p6. Two α -helices, H1 and H2, are represented by the light green bars. The binding domains for tumor susceptibility gene 101 (TSG101), ALG-2 interacting protein X (ALIX) and Vpr visually portrayed in dark blue, red, and purple, respectively. Phosphorylation sites are indicated by orange spheres. The attachment sites for ubiquitin (Ub) are depicted as light green spheres, while the attachments sites for small ubiquitin-like modifier 1 (SUMO-1) are designated by light blue spheres. The grey spheres correspond to the glutamic acids that contribute to viral replication. (**B)** HIV-1 budding process facilitated by Gag p6 and viral replication promoted by Vpr. The Gag p6 recruits endosomal sorting complex required for transport (ESCRT) to facilitate membrane scission and particle release. In subsequent infections, Vpr performs various functions, including promoting the reverse-transcription process, facilitating the nuclear import of the pre-integration complex, inducing cell cycle arrest and apoptosis, transactivating the HIV-LTR, and counteracting host restriction factors

To facilitate its own budding process, HIV-1 has evolved to exploit the cellular ESCRT machinery. This pathway has become a crucial mechanism employed by enveloped viruses to aid their own budding [11]. Two key proteins, TSG101 and ALIX, are often hijacked by viruses, including HIV-1, to promote viral replication and dissemination. Specifically, HIV-1 Gag p6 directly interacts with TSG101 and ALIX to recruit ESCRT complexes for viral assembly and budding [12–15]. The ^7^PTAPP^11^ motif within Gag p6, also known as the L domain, was first to be identified to facilitate viral assembly and budding processes by recruiting TSG101 [15]. On the other hand, the motif YPXnL in Gag p6 is involved in the binding of ALIX (Fig. 2A and B). Subsequently, ALIX links HIV-1 Gag to ESCRT-III, of which ESCRT-III in turn forms helical filaments at the neck of the bud and recruits the vacuolar protein sorting-associated protein 4 ATPase (VPS4 ATPase) to facilitate the final scission of virions from the plasma membrane [10, 16].

## The incorporation of Vpr mediated by Gag p6

HIV-1 Vpr is expressed during the late stage of the virus life cycle and is incorporated into newly formed viral particles by its interaction with the C-terminal domain of the Gag precursor, which includes HIV-1 nucleocapsid protein 7 (NCp7) and Gag p6 [17–20]. The zinc fingers of HIV-1 NCp7 directly interact with Vpr. However, this interaction has only been observed in vitro, and its role in the viral cycle remains to be explored. In contrast, Gag p6 alone is found to be sufficient for the incorporation of HIV-1 Vpr into heterologous viral particles [21, 22]. Furthermore, ^41^LXXL^44^ domain in Gag p6 is first identified to interact with Vpr. Mutational analysis has shown that motifs ^41^LXXLF^45^ at the C-terminus and ^15^FXFG^18^ at the N-terminus of Gag p6 are individually identified to be required for Vpr incorporation [18–20]. Moreover, mutations in the motif ^34^ELY^36^ of Gag p6 impair the recruitment of HIV-1 Vpr into viral particles [23]. It is worth noting that these binding sites within Gag p6 demonstrate a synergistic effect on the incorporation of HIV-1 Vpr when subjected to mutation [23, 24]. HIV-1 Vpr consists of three α-helices that combine to form a hydrophobic core. This hydrophobic core creates a hydrophobic plateau, which facilitates the formation of Vpr oligomers [25, 26]. Vpr oligomerization is crucial for its recognition by Gag p6 [24]. Solely inhibiting Vpr oligomerization results in a notable two-fold decrease in Vpr incorporation in the case of the L67A mutation, which hinders Vpr oligomerization. Furthermore, when Vpr oligomerization is disrupted together with removing either binding motif in Gag p6, the reduction in Vpr incorporation is approximately 25- to 50-fold greater compared to solely inhibiting Vpr oligomerization [23]. In addition, the high flexibility of HIV-1 Gag p6 suggests that it may not maintain a stable conformation in solution, thus posing challenges in investigating not only the binding sites of Gag p6 to HIV-1 Vpr but also its interactions with other partners. As a conclusion, no consensus has been well established on the residues or motifs essential for the interactions between Gag p6 and Vpr.

The incorporation of HIV-1 Vpr into virions mediated by Gag p6 facilitates viral replication in the newly infected cells by triggering “butterfly effects”, including regulation of viral reverse transcription, nuclear import of the pre-integration complex (PIC), transactivation of the HIV-1 long terminal repeat (LTR) promoter, and induction of cell cycle G2 arrest and apoptosis (Fig. 2B). In addition, extensive studies on HIV-1 have revealed the existence of intracellular restriction factors. Interestingly, HIV-1 Vpr can antagonize these restrictive factors through the utilization of the CUL4A-DDB1-DCAF1 ubiquitination system (Fig. 2B). This system involves an E3 ubiquitin ligase complex responsible for mediating protein ubiquitination and subsequent degradation through a proteasome-dependent pathway. The formation of the CUL4A-DDB1-DCAF1-Vpr complex enables HIV-1 Vpr to recruit restriction factors, promoting the polyubiquitination and degradation of these restriction factors. Here, we summarize several important restriction factors including synthetic lethal of unknown function 4 (SLX4), ten-eleven translocation 2 (TET2), human silencing hub (HUSH), uracil DNA glycosylase 2 (UNG2) and lysosomal-associated transmembrane protein 5 (LAPTM5) [27–31]. It’s important to note that the specific interactions and effects may vary depending on the specific strain of HIV-1.

## Other functions of Gag p6

Viral and cellular kinases play important roles in viral infections as they are involved in various cellular signal transduction processes. Among these processes, phosphorylation has been extensively studied as a post-translational modification that regulates numerous cellular functions. Interestingly, the phosphorylation of virus-encoded proteins plays an important role in viral protein stability, activity, interactions with other cellular components, as well as viral infection and replication. In the case of HIV-1, Gag p6 undergoes phosphorylation (Fig. 2A), with Ser and Thr serving as substrates for virion associated kinases [32]. Specifically, the phosphorylation of a highly conserved Ser residue at position 40 has been identified to be important for the infectivity of the released virions. The mutation of S40F in Gag p6 has been demonstrated to specifically disrupt the cleavage process between the CA and the spacer peptide SP1. Consequently, this disruption of CA processing leads to an irregular formation of the virus core, thereby affecting the maturation of viral particles [33]. This observation suggests that the phosphorylation of Gag p6 affects the CA maturation and viral core formation. Furthermore, the S40F mutation results in a significant reduction in infectivity, approximately 6 ~ 10-fold lower compared to the wild-type virus [33]. Therefore, blocking the function of Gag p6 may be potentially achieved by inhibiting enzymes involved in p6 phosphorylation.

Moreover, as a free form, HIV-1 p6 shows high affinity for membrane bilayers, which substantially increases its interaction with Vpr [8, 34]. Subsequent investigations have identified several glutamic acids within Gag p6 (Fig. 2A) that contribute to the interaction of Gag with the plasma membrane [14]. Notably, Gag p6 also regulates the process of the CA-SP1 cleavage, the assembly of the virus core, and the rate of defective ribosomal product formation and major histocompatibility complex (MHC) class I antigen presentation of Gag [33, 35]. In addition, Gag p6 can be covalently attached to ubiquitin and small ubiquitin-like modifier 1 (SUMO-1) [36, 37], which negatively impacts HIV-1 replication (Fig. 2A). However, the precise mechanism underlying this effect remains unclear.

## Gag p6 as a potential target for HIV-1 therapy

With the application of current antiretroviral therapy (ART), HIV infection is no longer considered a fatal disease. However, complete eradication of HIV in treated patients remains unachievable due to the existence of viral reservoirs within cells and tissues, despite the availability of antiretroviral drugs such as reverse transcriptase inhibitors, protease inhibitors, integrase inhibitors, and entry inhibitors [38]. Furthermore, the emergence of drug-resistant viruses and the adverse effects associated with each antiviral drug class pose challenges in improving treatment efficacy and reducing toxicity of ART. Therefore, it is necessary for the exploration of new targets to develop novel drugs.

The process of HIV-1 budding is dependent on the interactions between Gag p6 and TSG101, as well as Gag p6 and ALIX, indicating potential targets for the development of novel anti-HIV drugs that disrupt HIV budding. It has been observed that overexpressing the N-terminal domain of TSG101 effectively inhibits HIV-1 budding by blocking the ^7^PTAP^10^ domain function of Gag p6 [39]. Furthermore, several cyclic peptides with a primary sequence distinct from the interacting sites of both TSG101 and Gag p6 have been discovered to reduce virus-like particle release by disrupting the interaction between Gag p6 and TSG101 [40, 41]. Importantly, compounds such as hydrazone and hydrazide-containing N-substituted glycines exhibited higher binding affinity to TSG101 compared with Gag p6, thus potentially serving as HIV budding antagonists [42]. Consequently, these findings highlight the potential of blocking HIV budding by disrupting the interaction between Gag p6 and TSG101, which will lead to promising antiviral treatments.

The incorporation of HIV-1 Vpr into virion is mediated by Gag p6. In the newly infected cells, HIV-1 Vpr promotes viral replication by promoting viral gene transcription, reverse transcription, nuclear transport of the PIC, as well as degradation of host restriction factors. Vpr deletion mutant has been demonstrated to significantly slow down the progression of viral immunodeficiency symptoms in rhesus monkeys [43]. Furthermore, Vpr is required for viral replication in non-dividing cells. Disrupting the interaction of Gag p6 with Vpr will produce Vpr-defective viral particles. Consequently, this disruption would abolish all Vpr functions and significantly reduce viral replication in subsequent infections caused by Vpr-defective viral particles. Therefore, targeting the interaction between Gag p6 and Vpr is an attractive candidate for antiretroviral therapy, aiming to impede viral replication and reduce the progression of HIV infection.

The diverse functions of Gag p6 make it an attractive target for the drug development. However, only a limited number of inhibitors have been identified to disrupt the interaction of Gag p6 with its partners, as shown in Table 1. Several factors contribute to the limited research that has been done on HIV-1 p6 inhibitors. First, the development of inhibitors against HIV-1 is mainly focused on targeting viral enzymes, such as transcriptase, reverse transcriptase, integrase, and protease, which has resulted in less attention being paid to Gag p6. Second, the multifaceted roles of Gag p6 in viral replication present a challenge for the development of the inhibitors that specifically target this region without interfering with other essential processes. Moreover, the dynamic nature of Gag p6 and its varying conformations under different conditions further complicate the identification of binding sites of Gag p6 to its partners and challenge the development of small molecules that can effectively interact with Gag p6. Therefore, it is necessary to screen appropriate stabilizing agents to maintain a stable conformation of Gag p6, which will facilitate the identification of critical regions or binding sites where Gag p6 interacts with its partners. Additionally, these stabilizing agents not only maintain the structure of Gag p6 but also have the potential to block critical regions or binding sites of Gag p6 that interact with its partners, thereby acting as potential inhibitors. Furthermore, computational modeling techniques, such as fragment-based drug design and other computational methods, offer promising avenues for the development of Gag p6 inhibitors. As a result, ongoing research in this field will likely lead to the discovery of novel inhibitors against HIV-1.

**Table 1:** HIV-1 p6 inhibitors targeting viral budding

Inhibitors	Targeting sites	Antiviral mechanism	References
Cyclic peptide 11	UEV domain of TSG101	Inhibiting HIV budding by disrupting the interaction between Gag p6 and UEV domain of TSG101	[40]
Peptoid hydrazones and peptoid hydrazides			[42]
Cyclic peptide KRL74			[41]
Peptide XY3-3			[44]
Esomeprazole and tenatoprazole			[45]
TSG-5′ (TSG101 N-terminal with residues 10–240)	PTAP motif of Gag p6	Blocking the binding of TSG101 to Gag p6, thereby inhibiting HIV budding	[39]
Alix fragment (Residues 364–716)	YPXL motif of Gag p6	Inhibiting HIV budding by blocking the binding of Alix to Gag p6	[46]

## Conclusions

Gag p6, despite a small protein, serves as binding sites for various cellular and viral factors and plays a crucial role in the formation of infectious HIV-1 viruses. Importantly, several specific cyclic peptides have been identified as effective disruptors of the interaction between Gag p6 and TSG101. This disruption effectively inhibits HIV-1 budding and reduces the release of virus-like particles. These findings strengthen the potential for developing antiretroviral drugs that specifically target Gag p6 for HIV-1 therapeutic interventions. Overall, the intricate interplay between viral budding, the mediation of Vpr incorporation, phosphorylation, membrane binding, post-translational modification, and Gag p6 highlights its significance in viral protein function, viral replication, and the pathogenesis during HIV-1 infection. As a result, drug development efforts focusing on p6-targeted antiretrovirals are still anticipated to undergo substantial growth in the forthcoming years.

## Data Availability

No datasets were generated or analysed during the current study.
